# Healthcare resource utilization and costs of letermovir for cytomegalovirus-prophylaxis after allogeneic hematopoietic cell transplantation in a real-world, multi-centre study in Germany

**DOI:** 10.1007/s10198-025-01890-4

**Published:** 2026-02-16

**Authors:** Marie Engelhard, Sebastian M. Wingen-Heimann, Annika Y. Classen, Igor-Wolfgang Blau, Gesine Bug, Corinna Hebermehl, Sabrina Kraus, Olaf Penack, Andrés R. Rettig, Timo Schmitt, Torsten Steinbrunn, Daniel Teschner, Maria J. G. T. Vehreschild, Claudia Wehr, J. Janne Vehreschild, Sina M. Pütz

**Affiliations:** 1 Faculty of Medicine, Department I of Internal Medicine, University of Cologne, University Hospital Cologne, Cologne, Germany; 2 Institute of Translational Research, Cologne Excellence Cluster on Cellular Stress Responses in Aging-Associated Diseases (CECAD), Faculty of Medicine, University Hospital Cologne, University of Cologne, Cologne, Germany; 3 Department I of Internal Medicine, Center for Integrated Oncology Aachen Bonn Cologne Duesseldorf (CIO ABCD) and Excellence Center for Medical Mycology (ECMM), Faculty of Medicine), University Hospital Cologne, University of Cologne, Cologne, Germany; 4 University of Applied Sciences for Economics and Management (FOM), Cologne, Germany; 5 German Centre for Infection Research (DZIF), Partner Site Bonn-Cologne, Cologne, Germany; 6 Department of Hematology, Oncology and Tumorimmunology, Charité – Universitätsmedizin Berlin, corporate member of Freie Universität Berlin and Humboldt- Universität zu Berlin, Augustenburger Platz 1, 13353 Berlin, Germany; 7 Department of Medicine 2, Goethe University Frankfurt, University Hospital, Frankfurt am Main, Germany; 8 Department of Hematology and Medical Oncology, University Medical Centre of the Johannes Gutenberg University Mainz, Mainz, Germany; 9 Department of Internal Medicine II, University Hospital of Würzburg, Würzburg, Germany; 10 Berlin Institute of Health at Charité – Universitätsmedizin Berlin, Charitéplatz 1, 10117 Berlin, Germany; 11 Department of Medicine I/Hematology, Oncology and Stem Cell Transplantation, Medical Centre, Faculty of Medicine, University of Freiburg, University of Freiburg, Freiburg, Germany; 12 Department of Medical Oncology, Dana-Farber Cancer Institute, Harvard Medical School, Boston, MA USA; 13 Department of Internal Medicine, Infectious Diseases, Goethe University Frankfurt, University Hospital Frankfurt, Frankfurt am Main, Germany; 14 Faculty of Medicine, Goethe University Frankfurt, Institute for Digital Medicine and Clinical Data Science, Frankfurt am Main, Germany; 15 Department I of Internal Medicine, Division of Infectious Diseases, University Hospital Cologne, Kerpener Straße 62, 50937 Cologne, Germany

**Keywords:** Letermovir, Cytomegalovirus, Allogeneic hematopoietic cell transplantation, Healthcare resource utilization and costs, Real-world data

## Abstract

**Objectives:**

Letermovir has demonstrated efficacy and tolerability as prophylaxis against clinically significant cytomegalovirus infection (csCMVi) in CMV-seropositive adult patients undergoing allogeneic hematopoietic cell transplantation (alloHCT) compared to alternative antivirals. Despite these advantages, associated costs remain substantial. This study addresses the evidence gap by evaluating the health economic impact of CMV prophylaxis with letermovir in a real-world setting for alloHCT patients.

**Methods:**

This retrospective, multi-centre case-control study was conducted at six German tertiary care centres. A micro-costing approach evaluated hospitalisation and anti-CMV drug acquisition costs over a 48-week follow-up period post-alloHCT. The analysis included patients surviving at least 100 days following alloHCT, comparing individuals receiving letermovir prophylaxis (cases) with those who did not (controls) between January 2018 and April 2021.

**Results:**

The incidence of csCMVi was significantly higher in the control than in the letermovir group (56%, *n* = 98 vs. 34%, *n* = 63, *p* < 0.001). Median hospital length of stay was significantly longer in the control group (45 days, IQR 36–66) compared to the letermovir group (39 days, IQR 32–55; *p* < 0.001). Hospitalisation costs were comparable (*p* = 0.865), while anti-CMV drug acquisition and overall direct treatment costs were significantly higher in the letermovir group (€21,844, 95% CI 19,247 − 25,107 vs. €7,711, 95% CI 12,692 − 25,107, *p* < 0.001; 81,871, 95% CI 76,721 − 87,021 vs. 67,161, 95% CI 61,693 − 72,629, *p* < 0.001). Besides letermovir, cost drivers post-alloHCT were rehospitalisation and csCMVi.

**Conclusions:**

Our study demonstrated higher anti-CMV drug acquisition costs and overall direct treatment costs in patients receiving letermovir prophylaxis compared to controls. However, these higher costs are accompanied by significantly improved clinical outcomes.

**Supplementary Information:**

The online version contains supplementary material available at https://doi.org/10.1007/s10198-025-01890-4.

## Introduction

The anti-cytomegalovirus (CMV) prophylaxis letermovir was approved by the Food and Drug Administration (FDA) in November 2017 and by the European Medicines Agency (EMA) in January 2018 for prophylaxis of clinically significant CMV infections (csCMVi) in CMV-seropositive adult patients receiving allogeneic hematopoietic cell transplantation (alloHCT) [1]. Previously approved antiviral agents for anti-CMV prophylaxis or therapy are ganciclovir, valganciclovir, foscarnet, and cidofovir. Due to their association with adverse events such as nephrotoxicity and myelosuppressive side effects, effective and tolerable drugs for prevention and treatment of csCMVi were urgently needed [2]. Clinical studies have proven that prophylaxis with letermovir after alloHCT reduced csCMVi, including CMV end-organ disease or CMV viremia [3–8]. Moreover, letermovir use for this indication is associated with less side effects and resistance developments [2, 9, 10]. Studies evaluating the costs of pre-emptive CMV therapy in patients after alloHCT [10–12] have shown that a higher number of CMV episodes was associated with increased costs (12). Additionally, medical resource utilization and costs were higher in patients with csCMVi compared to those without csCMVi [11]. Health economic studies comparing CMV prophylaxis with letermovir vs. pre-emptive CMV therapy [4–6, 8] found that although the acquisition costs of letermovir prophylaxis were more expensive than costs of pre-emptive therapy, it was cost-effective due to increasing life-expectancy [5, 6, 8]. Clinical studies that examined the direct costs related to CMV infection and disease reported comparable results, but they included smaller sample sizes and shorter follow-up periods [4, 10, 12]. Multi-centre studies with larger study populations and longer study periods examining the economic burden of csCMVi in a real-world setting offer reliable and generalisable results needed to establish new, effective CMV preventative approaches [11]. In this multi-centre, observational study, we examined the healthcare resource utilization and costs of letermovir as prophylaxis of csCMVi for patients receiving alloHCT at six German university hospitals.

## Methods

### Study design and study population

A retrospective, multi-centre case-control study at six German university hospitals was performed. In the original study, 200 adult patients who received letermovir as primary CMV prophylaxis after alloHCT between January 2018 and April 2021 were included in the case group [13]. Those patients were compared to 200 matched control patients who did not receive letermovir prophylaxis. Patients in the control group received their alloHCT prior to approval of letermovir in Germany between January 2016 and December 2017. The follow up period was 48 weeks after alloHCT or until the patient´s death. Because of the health economic focus of this paper, only patients who survived at least 100 days following alloHCT were considered. The study was conducted in line with the Strengthening the Reporting of Observational Studies in Epidemiology (STROBE) statement, the Consolidated Health Economic Evaluation Reporting Standards 2022 (CHEERS), as well as national and international recommendations for health economic evaluation [14–17]. A completed CHEERS checklist is provided in Online Resource (Table S1). The study design, inclusion and exclusion criteria and matching factors have been described in more detail in the clinical paper from Hopff et al. [13].

### Definitions

CsCMVi was defined by the onset of CMV end-organ disease or initiation of anti-CMV pre-emptive therapy based on documented CMV viremia [3]. Breakthrough infections were defined as csCMVi appearing during CMV prophylaxis with letermovir. The index hospitalisation was considered the hospital stay during which the alloHCT was performed. Rehospitalisation was defined as one or more readmissions after discharge of the index hospital episode within the observation period. The overall hospital length of stay (LOS) was defined as the total number of hospitalisation days, including the initial hospital stay, during the 48-week observation period.

### Study objectives and hypotheses

The primary objective was to compare the overall direct treatment costs of patients receiving prophylaxis with letermovir vs. no prophylaxis with letermovir after alloHCT. In detail, costs of anti-CMV drug acquisition, diagnostic tests, and all hospitalisations during the observed period were calculated using a micro-costing approach and compared between both groups. Moreover, the association between the occurrence of csCMVi and the health economic burden in terms of direct treatment costs incurred during the hospital stay within the first year post-transplant (48 weeks starting on day of transplant, defined as day 0) were assessed and compared between groups.

### Data Documentation

Data documentation was performed anonymously by medical documentation specialists under supervision of experienced hematologists at the participating centres. All data were retrospectively documented into a web-based eCRF, accessible via www.clinicalsurveys.net, upon completing the 48-week observation period or after death of the patient. Data monitoring and query management was performed by the leading study site.

### Healthcare resource utilization and cost analysis

The healthcare resource utilization and cost analysis were performed from the perspective of the German healthcare system [16]. While the treatment costs for csCMVi and for underlying diseases are sometimes intertwined, cost factors without relation to csCMVi (e.g. surgery, anesthesiology) were excluded from the calculations. Costs for treatment on hospital ward were calculated based on the German Diagnosis Related Groups (G-DRG) systematic provided by Institute for the Hospital Renumeration System (InEK - Institut für das Entgeltsystem im Krankenhaus) and represented personnel and material costs from the years 2016 to 2021 [18]. The G-DRG reimbursement primarily depends on the base rate, which is adjusted annually to reflect general cost developments. Costs for anti-CMV drug acquisition were calculated per patient by using the pharmacy retail price from 2024 extracted from the Rote Liste, a comprehensive drug directory in Germany [19]. To ensure comparability of hospitalisation and CMV drug acquisition costs, the micro-costing approach used year 2024 values expressed in Euros (€). Indirect cost factors, such as productivity losses due to illness related disability or death before retirement age, were disregarded due to severity of hematological diseases.

### Statistical analysis

R Studio version 4.5.0 (from 2025-04-11) was used for data analysis. Patient characteristics and outcomes were presented as percentage (absolute numbers) for categorical variables and mean (95% confidence interval = 95% CI) for continuous variables. Age and treatment durations were described as median (interquartile range (IQR)/range). Treatment durations were calculated for patients receiving the respective drug. Statistical significance was indicated using the Pearson’s chi square test, Mann-Whitney U Test, log rank test or student’s t-test (two-sided), as appropriate, with *p* < 0.05 as significance level. For descriptive purposes, health economic data were presented as median (IQR/range) and/or mean (95% CI or standard deviation). Non-normally distributed cost values were analysed and compared by using Welch´s bootstrapped t-test, whereby bootstrapping were performed by using 10,000 samples and the starting point for the Mersenne twister was at 1,000. Moreover, a multivariate generalized linear model (GLM) with gamma distribution and log-link was applied to analyse variables influencing overall direct treatment costs. The GLM reported Odds Ratios (OR) as well as 95% CIs. Prior to GLM, a univariate regression analysis was performed to identify variables for inclusion into the model. Predictors with a significance level of p = < 0.1 were evaluated in the GLM.

## Results

### Study population

In the letermovir and control group, 186 and 176 patients survived at least 100 days after alloHCT and were therefore analysed in this study. Patients in the letermovir group were significantly older than those in the control group with a median age of 60 years compared to 56 years (*p* = 0.041). Regarding the other baseline characteristics of both groups, no significant differences were found (Table 1). In the clinical analysis from Hopff et al., a more detailed table of patient characteristics is presented [13]. The incidence of csCMVi was significantly higher in the control than in the letermovir group with 56% (*n* = 98) vs. 34% (*n* = 63, *p* < 0.001). In the letermovir group, in 49% (*n* = 31) of patients, csCMVi occurred after, in 40% (*n* = 25) during and in 11% (*n* = 7) before letermovir prophylaxis. Among the patients that survived at least 100 days, 22% (*n* = 41) in the letermovir group and 28% (*n* = 50) in the control group died within the 48-week observation period (*p* = 0.163). Among those, 39% (*n* = 16) and 58% (*n* = 29) had csCMVi, respectively (*p* = 0.072). There were no significant differences regarding csCMVi as a contributing factor to the cause of death in the letermovir (2%; *n* = 1) and control group (14%; *n* = 7; *p* = 0.236).

**Table 1:** Characteristics of alloHCT patients surviving at least day 100 post-transplant

	Letermovir group (*n* = 186)	Control group (*n* = 176)	*p* value	
Age in years; Median (Range)	60 (18–77)	56 (18–77)	0.041^a^*	
Female gender; n (%)	80 (43)	73 (42)	0.768^b^	
Ethnic origin; n (%)			0.529^b^	
Caucasian	183 (98)	171 (97)		
Asian	3 (2)	4 (2)		
Hispanic/Latino	-	1 (1)		
Body mass index; Mean (95% CI)	25.3 (24.1–26.4)	26.4 (25.3–27.4)	0.174^c^	
Primary underlying disease; n (%)	186 (100)	176 (100)	0.260^b^	
Acute myeloid leukaemia	90 (48)	97 (55)		
Myelodysplastic syndrome	24 (13)	20 (11)		
Non-Hodgkin-Lymphoma	20 (11)	8 (5)		
Acute lymphoblastic leukaemia	18 (10)	17 (10)		
Chronic myeloid leukaemia	7 (4)	4 (2)		
Other	27 (15)	30 (17)		
GvHD; n (%)	85 (46)	91 (52)		
Grade 1	64 (34)	57 (32)	0.684^b^	
Grade 2	12 (7)	24 (14)	0.022^b^*	
Grade 3	6 (3)	10 (6)	0.256^b^	
Grade 4	3 (2)	0 (0)	0.249^d^	
No GvHD	101 (54)	85 (48)	0.253^b^	
csCMV infection; n (%)			< 0.001^b^***	
CMV disease, n (%)	-	4 (2)		
csCMV replication in blood (viremia) warranting antiviral treatment	63 (34)	94 (53)		
No csCMV replication/CMV disease occurred	123 (66)	78 (44)		

*CI* confidence interval, *CMV* cytomegalovirus, *csCMV* clinically significant cytomegalovirus, *GvHD* Graft-versus-Host-Disease, IQR interquartile range

* p-value < 0.05; *** p-value < 0.001

^a^ Mann-Whitney U-test

^b^ Pearson chi-square test (two-tailed)

^c^ Student t-test

^d^ Fisher´s exact test

### Hospitalisation and treatment

The median length of the initial hospitalisation was significantly longer in the control group with 38 days (IQR 32–45 days) compared to the letermovir group with 35 days (IQR 31–42 days, *p* = 0.046). The same trend could be observed for the overall median hospital LOS (control group: median 45 days, IQR 36–66 days vs. letermovir group: median 39 days, IQR 32–55 days, *p* < 0.001). Number of hospital admissions (letermovir group: range 1–6, control group: range 1–8, *p* = 0.165) and the frequency of patients with rehospitalisations (letermovir group: 28%, *n* = 52, control group: 35%, *n* = 61, *p* = 0.169) were comparable. 8% (*n* = 15) of the letermovir group and 10% (*n* = 17) of the control group were treated on ICU (*p* = 0.593). Patients in the control group had a significantly longer stay on the intermediate care unit (letermovir group: median 6 days, IQR 4–19 days, control group: median 32 days, IQR 22–41 days, *p* = 0.017). The LOS on the other wards was comparable between both groups (Table 2).

**Table 2:** Hospitalisation and CMV prophylaxis and treatment details

	Letermovir group (*n* = 186)	Control group (*n* = 176)	*p* value	
Hospitalisation; n (%)	186 (100)	176 (100)	-	
Length of initial hospitalisation (days); Median (IQR)	35 (31–42)	38 (32–45)	0.046^a^*	
Overall LOS (days); Median (IQR)	39 (32–55)	45 (36–66)	< 0.001^a^***	
Number of hospitalisations; Median (Range)	1 (1–6)	1 (1–8)	0.165^a^	
Rehospitalisations; n (%)	52 (28)	61 (35)	0.169^b^	
1 rehospitalisation	24 (13)	29 (17)	0.336^b^	
≥ 2 rehospitalisation	28 (15)	32 (18)	0.424^b^	
Stay on hospital wards				
Bone marrow transplant ward				
Total days; Median (IQR)	37 (32–51)	41 (33–53)	0.291^a^	
General ward				
Total days; Median (IQR)	15 (6–31)	13 (8–38)	0.484^a^	
Intermediate care unit				
Total days; Median (IQR)	6 (4–19)	32 (22–41)	0.017^a^*	
Intensive care unit				
Total days; Median (IQR)	4 (2–10)	5 (2–7)	0.703^a^	
Mechanical ventilation				
Total hours; Median (IQR)	84 (55–126)	312 (162–402)	0.054^a^	
Prophylaxis and Treatment				
Letermovir^I^; n (%)	183 (98)	-	-	
Duration (days); Median (IQR)	94 (77–101)	-	-	
Ganciclovir; n (%)	29 (16)	59 (34)	< 0.001^b^***	
Duration (days); Median (IQR)	9 (7–14)	14 (8–22)	< 0.001^a^***	
Valganciclovir; n (%)	31 (17)	51 (29)	0.005^b^**	
Duration (days); Median (IQR)	13 (5–24)	15 (5–28)	0.431^a^	
Foscarnet; n (%)	12 (7)	46 (26)	< 0.001^b^***	
Duration (days); Median (IQR)	11 (8–16)	17 (11–22)	0.039^a^*	
Cidofovir; n (%)	3 (2)	5 (3)	0.492^c^	
Duration (days); Median (IQR)	5 (5–13)	28 (7–44)	0.371^a^	

*CI* confidence interval, *IQR* interquartile range, *LOS* length of stay

* p-value < 0.05; ** p-value < 0.01; *** p-value < 0.001

^I^ n-values differ due to missing data

^a^ Mann-Whitney U-test

^b^ Pearson chi-square test (two-tailed)

^c^ Fisher´s exact test

In the letermovir group, treatment with letermovir started at a median of ten days after alloHCT (IQR 2–21 days). All patients received letermovir orally for a median duration of 94 days (IQR 77–101 days). For 2% (*n* = 3) in the letermovir group, no data were provided for the duration and dosage of letermovir, resulting in their exclusion from the cost analysis for letermovir. If a csCMVi occurred, patients received an anti-CMV therapy with ganciclovir, valganciclovir, cidofovir, or foscarnet, as described in Table 2. To enhance comparability with other studies in this field and to provide a comprehensive understanding of treatment exposure, treatment durations were also calculated per patient (Table S1).

### Costs for hospitalisation and CMV treatment

Direct treatment cost factors are described in Table 3. When applying the values of the year 2024, hospitalisation costs were comparable between both groups with a mean of €59,772 per patient (95% CI 56,000–63,545) in the letermovir and €59,371 per patient (95% CI 55,540–63,203) in the control group (*p* = 0.865). Treatment costs with letermovir amounted to an average of €21,070 per patient (95% CI 18,526 − 24,281). Regarding the other anti-CMV agents, mean costs for ganciclovir and foscarnet were significantly higher in the control group than in the letermovir group (*p* < 0.001; *p* < 0.001). Overall anti-CMV drug acquisition costs as well as overall direct treatment costs were significantly higher in the letermovir than in the control group (€21,844, 95% CI 19,247–25,107 vs. €7,711, 95% CI 5,407–10,408, *p* < 0.001; €81,871, 95% CI 76,721 − 87,021 vs. €67,161, 95% CI 61,693–72,629, *p* < 0.001). Regarding the subgroup of patients with csCMVi, average costs for hospitalisation were significantly higher in the control group (*p* < 0.001), whereas drug acquisition costs were higher in the letermovir group (*p* = 0.003). Overall direct costs were comparable between both groups (*p* = 0.239). In patients without csCMVi, all direct cost parameters were significantly higher in the letermovir group. In the letermovir group, patients with csCMVi incurred 10% lower overall costs compared to those without csCMVi, whereas in the control group, patients with csCMVi resulted in 43% higher overall costs. Detailed information on this are given in the Online Resource (Table S3, Table S4).

**Table 3:** Direct treatment costs for patients after alloHCT in Euro

	Letermovir group (*n* = 186)	Control group (*n* = 176)	*p* value
Hospitalisation costs^I, b^; n (%)	181 (97)	173 (98)	0.724^d^
Costs per patient; mean (95% CI)	61,882 (57,727 − 66,036)	76,507 (72,374 − 80,640)	< 0.001^a^***
Hospitalisation costs^I^; n (%) (Year 2024)	181 (97)	173 (98)	0.724^d^
Costs per patient; mean (95% CI)	59,772 (56,000–63,545)	59,371 (55,540 − 63,203)	0.865^a^
Anti-CMV drug acquisition costs^c^; n (%)	186 (100)	176 (100)	-
Costs per patient; mean (95% CI)			
Letermovir	21,070 (18,526 − 24,281)	-	-
Ganciclovir	327 (188–497)	1,224 (829-1,682)	< 0.001^a^***
Valganciclovir	636 (308-1,077)	897 (490-1,411)	0.376^a^
Foscarnet	582 (221-1,052)	5,478 (3,486-7,905)	< 0.001^a^***
Cidofovir	23 (0–55)	112 (8–247)	0.101^a^
Overall anti-CMV drug acquisition costs^c^; n (%)	186 (100)	176 (100)	-
Overall costs per patient; mean (95% CI)	21,844 (19,247 − 25,107)	7,711 (5,407 − 10,408)	< 0.001^a^***
Overall direct treatment costs^I, b, c^; n (%)	181 (97)	173 (98)	0.724^d^
Overall costs per patient; mean (95% CI)	83,981 (78,605 − 89,357)	84,296 (78,526 − 90,067)	0.941^a^
Overall direct treatment costs^I^; n (%) (Year 2024)	181 (97)	173 (98)	0.724^d^
Overall costs per patient; mean (95% CI)	81,871 (76,721 − 87,021)	67,161 (61,693 − 72,629)	< 0.001^a^***

*CI* Confidence interval, *CMV* cytomegalovirus

*** p-value < 0.001

^I^ n-values differ due to missing data

^a^ Bootstrapped t-test (independent samples, two sided)

^b^ Based on G-DRGs from 2016 to 2021

^c^ Based on Rote Liste prices from 2024

^d^ Fisher´s exact test

The GLM demonstrated predictors influencing overall direct treatment costs of patients undergoing alloHCT (Table 4). The variables with the highest impact on costs were rehospitalisation (OR 1.79, *p* < 0.001), being a patient in the letermovir group (OR 1.39, *p* < 0.001), and csCMVi (OR 1.10, *p* = 0.010). Patients receiving immunosuppressive agents were found to be associated with significantly lower overall direct treatment costs (OR 0.60; *p* < 0.001). Treatment on intensive care unit did not remain significant in the multivariate GLM (*p* = 0.05), although borderline significant. Age, gender, acute leukaemia and occurrence of acute GvHD grade II-IV were found to not influence the overall treatment costs of the study cohort.

**Table 4:** Generalized linear model of variables influencing overall treatment costs of patients after alloHCT

Overall treatment costs	Univariate GLM			Multivariate GLM		
	OR	95% CI	*p*	OR	95% CI	*p*
Age	0.998	0.995–1.002	0.421	-	-	-
Gender (Reference = Female)						
Male	1.059	0.956–1.173	0.272	-	-	-
Groups (Reference = Control group)						
Letermovir group	1.219	1.101–1.349	< 0.001***	1.389	1.293–1.492	< 0.001***
Acute leukaemia	1.088	0.981–1.207	0.111	-	-	-
Occurrence of acute GvHD ≥ grade II	0.960	0.861–1.073	0.470	-	-	-
csCMVi	1.148	1.036–1.272	0.009**	1.102	1.021–1.190	0.010*
Treatment on intensive care unit	1.297	1.097–1.546	0.003**	1.128	1.002–1.274	0.050
Rehospitalisation	1.641	1.501–1.795	< 0.001***	1.792	1.653–1.943	< 0.001***
Immunosuppressive therapy	0.740	0.632–0.862	< 0.001***	0.596	0.536–0.662	< 0.001***

*n* = 354; Akaike information criterium:8,125; Bayes information criterium: 8,152; deviation: 40.7; likelihood quotient chi-square: 240.5 (*p* < 0.001)

*CI* confidence interval, *csCMVi* clinically significant cytomegalovirus infection, *GLM* generalized linear model, *GvHD* graft-versus-host disease, *OR* Odds ratio

* p-value < 0.05; ** p-value < 0.01; *** p-value < 0.001

## Discussion

In this retrospective multi-centre observational study, healthcare resource utilization and costs of letermovir as CMV prophylaxis in patients receiving alloHCT in Germany were analysed. We found that csCMVi incidence and overall hospital LOS were significantly higher in patients in the control group compared to the letermovir group during the 48-week follow-up. While hospitalisation costs were comparable in both groups, costs for anti-CMV drug acquisition and overall treatment costs were significantly higher in the letermovir group. Identified cost drivers were rehospitalisation, being in the letermovir group and csCMVi.

The clinical outcome of letermovir-treated patients was significantly better with regard to the incidence of csCMVi than the one of patients treated with or without other anti-CMV agents which is in line with current studies [3, 5–8, 20]. In the phase 3 clinical trial from Marty et al., 19% of patients in the letermovir group had csCMVi compared to 50% of patients in the placebo group [3]. Sepassi et al. came to the conclusions that patients receiving letermovir prophylaxis had fewer CMV-viremia (16/100 vs. 40/100) compared to patients receiving exclusively pre-emptive therapy [6]. In our analysis, the frequency of rehospitalisations was comparable between the groups, while other current studies came to contrary results [5, 6, 8]. In the study from Sepassi et al., which was conducted in the United States, patients receiving letermovir prophylaxis had fewer CMV-related rehospitalisations (3/100 vs. 8/100) than patients receiving exclusively pre-emptive therapy [6]. Furthermore, the overall hospital LOS in our study population was significantly longer in the control group, but resulted in similar hospitalisation costs. The study from Tan et al. conducted in the United States described similar findings regarding the overall hospital LOS and different findings with lower hospitalisation costs. Patients receiving letermovir had a median LOS of 36 days (IQR 28–46) compared to patients receiving pre-emptive therapy with a median LOS of 38 days (IQR 29–64; *p* = 0.250) at a 180 day follow-up after alloHCT [21]. The hospitalisation costs were significantly higher in the group receiving pre-emptive therapy with a median of $ (US Dollar) 34,524 (IQR 20,904 − 57,201) compared to the letermovir group with a median of $10,930 (IQR 6,792 − 20,070; *p* < 0.001). We found that mean overall direct treatment costs for alloHCT patients were €81,871 per patient in the letermovir group and €67,161 per patient in the control group. Sepassi et al. reported comparable results with $83,857 per patient in the letermovir group and $70,661 per patient receiving pre-emptive therapy alone over a 10-year time horizon [6]. The cohort study from Tan et al. reported significantly higher total direct CMV-associated therapy costs in the letermovir group (median $42,629; IQR 31,214–42,752) than in the control group (median $9,056; IQR 0–22,612; *p* < 0.001) [21]. In addition to letermovir itself, we identified rehospitalisations and csCMVi as significant cost drivers across the study cohort, which may indicate that these patients required more complex treatment. In contrast to our findings, other studies have found that acute GvHD of grades II-IV were associated with higher overall costs [12, 22–24]. Because there were no significant differences regarding rehospitalisations between the groups, it can be assumed that letermovir itself and csCMVi were the most influential cost factors. Nonetheless, we could not identify which complications contributed most to rehospitalisation in each group because we did not collect specific reasons for readmission. This result is in line with the study from Robin et al. who concluded that the occurrence of two or more CMV episodes increases the cost of alloHCT by 25–30%, representing €25,000–30,000 [12]. Therefore, CMV prophylaxis with letermovir has the potential to reduce CMV episodes and associated costs. Additionally, when regarding hospitalisation costs in our cohort, the group of control patients with csCMVi incurred higher average costs compared to the group of letermovir patients with csCMVi. Nevertheless, when regarding the hospitalisation costs among alloHCT patients without csCMVi in our study, costs were significantly higher in letermovir-treated patients compared to control patients, suggesting that patients in this group may be generally sicker.

It should be noted that, although our study groups were generally well-matched, the potential influence of unknown confounding factors cannot be excluded and may have contributed to the observed increase in treatment costs. When comparing CMV treatment costs, it is important to consider that letermovir is still under patent and costs are expected to decrease once the patent expires. Also, letermovir is reimbursed as an additional payment (Zusatzentgelt) on top of the standard DRG-based reimbursement in Germany, which can increase its clinical use and reduce costs for hospitals. In a multicentre, randomised, placebo-controlled phase 3 trial, the efficacy and safety of extending CMV prophylaxis with letermovir to 200 days after allo-HSCT for high-risk patients were proven [25]. Consequently, higher treatment costs of extended CMV therapy, along with better clinical outcomes, such as the prevention of csCMVi and associated costs, must be considered when evaluating the health economic burden of letermovir in this population. Due to the retrospective design of our study it was not possible to retrieve data regarding the quality of life of the individual patients. Nevertheless, there are studies that demonstrated cost-effectiveness of letermovir for CMV-prophylaxis in alloHCT patients with improved health-related quality of life. Accordingly, letermovir prophylaxis increased life-years per person and quality adjusted life years (QALYs), resulting in an incremental cost-effectiveness ratio (ICER) of $59,356 per QALY gained [6]. In the study from Restelli et al. which was conducted from the perspective of the Italian NHS, prophylaxis with letermovir increased medical costs by €10,222 per patient and by 0.45 QALYs, leading to a cost-effectiveness ratio of 22,564 euro/QALY compared with no prophylaxis [5].

Our study is limited by its retrospective case-control study design, which prevents us from ruling out temporal trends that may have influenced the results. Also, when interpreting the results, one has to consider the temporal disparity of one to five years between the alloHCT of the letermovir and control group. To address this issue and ensure comparability of results, prices of G-DRGs and CMV drug acquisition from 2024 were used. Despite the GLM for identification of predictors influencing overall costs, the authors cannot rule out that variables outside the scope of this study may have influenced the results. Another limitation of this analysis is that the overall treatment costs did not account for additional procedures or examinations, which may lead to increased costs due to higher resource utilization in patients with csCMVi. Moreover, concomitant infections and their associated treatment costs related to CMV episodes were not explicitly considered. However, these were partially reflected in the hospital LOS and G-DRGs. The strength of our study lies in its multi-centre, matched cohort design conducted in a real-world setting, which ensures comparability between the groups and includes a larger sample size compared to other studies in this field [4, 12, 21]. Also, the follow-up period of 48 weeks is longer than in other studies, allowing long-term outcomes to be assessed. To our knowledge, this is the first study using a micro-costing approach on an individual level in a multi-centre real world-setting in Germany.

In conclusion, our study indicates that patients after alloHCT receiving letermovir for CMV-prophylaxis result in higher overall direct treatment costs compared to patients receiving other anti-CMV agents, while achieving improved clinical outcomes. Next to letermovir itself, rehospitalisation and csCMVi have been identified as significant cost drivers in the German inpatient setting. Future studies and clinical practice should focus on strategies to reduce CMV occurrence and rehospitalisation rates in CMV-seropositive patients undergoing alloHCT.

## Supplementary Information

Below is the link to the electronic supplementary material.

Supplementary Material 1 (DOCX 1.8 KB)

## Data Availability

The authors are not permitted to disclose the study data beyond the research team. However, the data can be accessed through direct request to the corresponding author.

## Materials availability

R scripts used to analyse the data can be made available on reasonable request.
