# Supplementary material for: Healthcare resource utilization and costs of letermovir for cytomegalovirus-prophylaxis after allogeneic hematopoietic cell transplantation in a real-world, multi-centre study in Germany
Source: Eur J Health Econ. 2026 Feb 16;27(7):1469–78. doi: 10.1007/s10198-025-01890-4 (PMC13619717; doi:10.1007/s10198-025-01890-4)
Supplement: Supplementary file 1 [file 10198_2025_1890_MOESM1_ESM.docx]

**Supplementary Material**

**Article title:** Healthcare resource utilization and costs of letermovir for cytomegalovirus-prophylaxis after allogeneic hematopoietic cell transplantation in a real-world, multi-centre study in Germany

**Journal:** European Journal of Health Economics

**Authors:** Marie Engelhard, Sebastian M. Wingen-Heimann, Annika Y. Classen^,^ Igor-Wolfgang Blau, Gesine Bug, Corinna Hebermehl, Sabrina Kraus, Olaf Penack, Andrés R. Rettig, Timo Schmitt, Torsten Steinbrunn^,^ Daniel Teschner, Maria J.G.T. Vehreschild, Claudia Wehr, J. Janne Vehreschild, and Sina M. Pütz

**Corresponding author:** Sina M. Pütz, University Hospital Cologne, Department I of Internal Medicine, Division of Infectious Diseases, Kerpener Straße 62, 50937 Cologne, Germany. E-mail address: [sina.hopff@uk-koeln.de](mailto:sina.hopff@uk-koeln.de)

Table S1: CHEERS checklist

| **Topic** | **No.** | **Item** | **Location where item is reported** |
| --- | --- | --- | --- |
| **Title** | | | |
|  | 1 | Identify the study as an economic evaluation and specify the interventions being compared. | Title |
| **Abstract** | | | |
|  | 2 | Provide a structured summary that highlights context, key methods, results, and alternative analyses. | Summary |
| **Introduction** | | | |
| **Background and objectives** | 3 | Give the context for the study, the study question, and its practical relevance for decision making in policy or practice. | Introduction |
| **Methods** | | | |
| **Health economic analysis plan** | 4 | Indicate whether a health economic analysis plan was developed and where available. | Healthcare resource and cost analysis |
| **Study population** | 5 | Describe characteristics of the study population (such as age range, demographics, socioeconomic, or clinical characteristics). | Study design and study population, Table 1 |
| **Setting and location** | 6 | Provide relevant contextual information that may influence findings. | Study design and study population |
| **Comparators** | 7 | Describe the interventions or strategies being compared and why chosen. | Study design and study population |
| **Perspective** | 8 | State the perspective(s) adopted by the study and why chosen. | Healthcare resource and cost analysis |
| **Time horizon** | 9 | State the time horizon for the study and why appropriate. | Study design and study population |
| **Discount rate** | 10 | Report the discount rate(s) and reason chosen. | Healthcare resource and cost analysis, Statistical analysis |
| **Selection of outcomes** | 11 | Describe what outcomes were used as the measure(s) of benefit(s) and harm(s). | Not Applicable |
| **Measurement of outcomes** | 12 | Describe how outcomes used to capture benefit(s) and harm(s) were measured. | Not Applicable |
| **Valuation of outcomes** | 13 | Describe the population and methods used to measure and value outcomes. | Not Applicable |
| **Measurement and valuation of resources and costs** | 14 | Describe how costs were valued. | Healthcare resource and cost analysis |
| **Currency, price date, and conversion** | 15 | Report the dates of the estimated resource quantities and unit costs, plus the currency and year of conversion. | Healthcare resource and cost analysis |
| **Rationale and description of model** | 16 | If modelling is used, describe in detail and why used. Report if the model is publicly available and where it can be accessed. | Not Applicable |
| **Analytics and assumptions** | 17 | Describe any methods for analysing or statistically transforming data, any extrapolation methods, and approaches for validating any model used. | Statistical Analysis |
| **Characterising heterogeneity** | 18 | Describe any methods used for estimating how the results of the study vary for subgroups. | Not applicable |
| **Characterising distributional effects** | 19 | Describe how impacts are distributed across different individuals or adjustments made to reflect priority populations. | Not applicable |
| **Characterising uncertainty** | 20 | Describe methods to characterise any sources of uncertainty in the analysis. | Statistical Analysis |
| **Approach to engagement with patients and others affected by the study** | 21 | Describe any approaches to engage patients or service recipients, the general public, communities, or stakeholders (such as clinicians or payers) in the design of the study. | Not Applicable |
| **Results** | | | |
| **Study parameters** | 22 | Report all analytic inputs (such as values, ranges, references) including uncertainty or distributional assumptions. | Not Applicable |
| **Summary of main results** | 23 | Report the mean values for the main categories of costs and outcomes of interest and summarise them in the most appropriate overall measure. | Costs for hospitalisation and CMV treatment |
| **Effect of uncertainty** | 24 | Describe how uncertainty about analytic judgments, inputs, or projections affect findings. Report the effect of choice of discount rate and time horizon, if applicable. | Costs for hospitalisation and CMV treatment |
| **Effect of engagement with patients and others affected by the study** | 25 | Report on any difference patient/service recipient, general public, community, or stakeholder involvement made to the approach or findings of the study | Not Applicable |
| **Discussion** | | | |
| **Study findings, limitations, generalisability, and current knowledge** | 26 | Report key findings, limitations, ethical or equity considerations not captured, and how these could affect patients, policy, or practice. | Discussion |
| **Other relevant information** | | | |
| **Source of funding** | 27 | Describe how the study was funded and any role of the funder in the identification, design, conduct, and reporting of the analysis | Funding |
| **Conflicts of interest** | 28 | Report authors conflicts of interest according to journal or International Committee of Medical Journal Editors requirements. | Declaration of Competing Interest |

Table S2: CMV prophylaxis and treatment durations per patient

|  | **Letermovir group (n=186)** | **Control group (n=176)** | **p value** |
| --- | --- | --- | --- |
| **Prophylaxis and Treatment; n (%)** | 186 (100) | 176 (100) | - |
| **Durations per patient (days); Median (IQR)** |  |  |  |
| **Letermovir** | 96 (80-104) | - | - |
| **Ganciclovir** | 0 (0-0) | 0 (0-8) | <0.001^a^*** |
| **Valganciclovir** | 0 (0-0) | 0 (0-2) | 0.004^a^** |
| **Foscarnet** | 0 (0-0) | 0 (0-4) | <0.001^a^*** |
| **Cidofovir** | 0 (0-0) | 0 (0-0) | 0.421^a^ |
| Abbreviations: IQR, interquartile range | | | |
| ** p-value <0.01; *** p-value <0.001 | | | |
| ^a^ Mann-Whitney U-test | | | |

Table S3: Direct treatment costs for patients after alloHCT with csCMVi in Euro

|  | **Letermovir group (n=63)** | **Control group (n=98)** | **p value^a^** | |
| --- | --- | --- | --- | --- |
| **Hospitalisation costs^I, b^; n (%)** | 61 (97) | 96 (98) | 0.645^e^ | |
| Costs per patient;  mean (95% CI) | 53,536 (47,639-59,434) | 87,004 (81,328-92,680) | <0.001*** | |
| **Hospitalisation costs^I^; n (%)**  **(Year 2024)** | 61 (97) | 96 (98) | 0.645^e^ | |
| Costs per patient;  mean (95% CI) | 52,234 (46,788-57,860) | 68,992 (63,672-74,313) | <0.001*** | |
| **Anti-CMV drug acquisition costs^c^; n (%)** | 63 (100) | 98 (100) | - | |
| Costs per patient;  mean (95% CI) |  |  |  | |
| Letermovir | 21,186 (17,569-25,402) | - | - | |
| Ganciclovir | 898 (531-1,338) | 2,137 (1,494-2,904) | 0.022* | |
| Valganciclovir | 1,743 (866-2,916) | 1,601 (889-2,498) | 0.852 | |
| Foscarnet | 1,670 (657-2,950) | 9,791 (6,345-13,833) | 0.001*** | |
| Cidofovir | 67 (0-161) | 202 (18-438) | 0.220 | |
| **Overall anti-CMV drug acquisition costs^c^; n (%)** | 63 (100) | 98 (100) | - | |
| Overall costs per patient;  mean (95% CI) | 23,883 (19,876-28,188) | 13,730 (9,915-18,262) | 0.003** | |
| **Overall direct treatment costs^I, b, c^; n (%)** | 61 (97) | 96 (98) | 0.645^e^ | |
| Overall costs per patient;  mean (95% CI) | 77,774 (69,981-85,568) | 101,000 (92,555-109,288) | <0.001*** | |
| **Overall direct treatment costs^I^; n (%) (Year 2024)** | 61 (97) | 96 (98) | 0.645^e^ | |
| Overall costs per patient;  mean (95% CI) | 76,562 (69,032-84,092) | 82,909 (74,922-90,896) | 0.239 | |
| Abbreviations: CI, Confidence interval; CMV, cytomegalovirus | | | |  |
| * p-value <0.05; ** p-value <0.01; *** p-value <0.001 | | | |  |
| ^I^ n-values differ due to missing data | | | |  |
| ^a^ Bootstrapped t-test (independent samples, two sided) | | | |  |
| ^b^ Based on G-DRGs from 2016 to 2021 | | | |  |
| ^c^ Based on Rote Liste prices from 2024 | | | |  |
| ^d^ Pearson chi-square test (two-tailed) | | | |  |
| ^e^ Fisher´s exact test | | | |  |

Table S4: Direct treatment costs for patients after alloHCT without csCMVi in Euro

|  | **Letermovir group (n=123)** | **Control group (n=78)** | **p value^a^** |
| --- | --- | --- | --- |
| **Hospitalisation costs^I, b^; n (%)** | 120 (98) | 77 (99) | 1.000^e^ |
| Costs per patient;  mean (95% CI) | 66,124 (60,725-71,524) | 63,419 (58,751-68,087) | 0.441 |
| **Hospitalisation costs^I^; n (%)**  **(Year 2024)** | 120 (98) | 77 (99) | 1.000^e^ |
| Costs per patient;  mean (95% CI) | 63,559 (58,701-68,416) | 47,376 (43,125-51,627) | <0.001*** |
| **Anti-CMV drug acquisition costs^c^; n (%)** | 123 (100) | 78 (100) | - |
| Costs per patient;  mean (95% CI) |  |  |  |
| Letermovir | 21,014 (17,838-25,448) | - | - |
| Ganciclovir | 35 (0-90) | 76 (0-181) | 0.442 |
| Valganciclovir | 69 (0-207) | 12 (3-23) | 0.740 |
| Foscarnet | 24 (0-72) | 60 (0-181) | 0.525 |
| **Overall anti-CMV drug acquisition costs^c^; n (%)** | 123 (100) | 78 (100) | - |
| Overall costs per patient; mean (95% CI) | 20,800 (17,603-25,118) | 149 (21-318) | <0.001*** |
| **Overall direct treatment costs^I, b, c^; n (%)** | 120 (98) | 77 (99) | 1.000^e^ |
| Overall costs per patient;  mean (95% CI) | 87,136 (80,053-94,219) | 63,570 (58,857-68,283) | <0.001*** |
| **Overall direct treatment costs^I^; n (%) (Year 2024)** | 120 (98) | 77 (99) | 1.000^e^ |
| Overall costs per patient;  mean (95% CI) | 84,570 (77,789-91,351) | 47,527 (43,229-51,825) | <0.001*** |
| Abbreviations: CI, Confidence interval; CMV, cytomegalovirus | | | |
| *** p-value <0.001 | | | |
| ^I^ n-values differ due to missing data | | | |
| ^a^ Bootstrapped t-test (independent samples, two sided) | | | |
| ^b^ Based on G-DRGs from 2016 to 2021 | | | |
| ^c^ Based on Rote Liste prices from 2024 | | | |
| ^d^ Pearson chi-square test (two-tailed) | | | |
| ^e^ Fisher´s exact test | | | |
